# Undercarboxylated Osteocalcin: Experimental and Human Evidence for a Role in Glucose Homeostasis and Muscle Regulation of Insulin Sensitivity

**DOI:** 10.3390/nu10070847

**Published:** 2018-06-29

**Authors:** Xuzhu Lin, Tara C. Brennan-Speranza, Itamar Levinger, Bu B. Yeap

**Affiliations:** 1 Institute of Health and Sport, Victoria University, Melbourne, VIC 8011, Australia; xuzhu.lin@live.vu.edu.au; 2 Department of Physiology, Bosch Institute for Medical Research, University of Sydney, Sydney, NSW 2006, Australia; tara.speranza@sydney.edu.au; 3 Australian Institute for Musculoskeletal Science, Department of Medicine, Western Health, Melbourne Medical School, University of Melbourne, Melbourne, VIC 3021, Australia; 4 The Medical School, University of Western Australia, Perth, WA 6009, Australia; 5 Department of Endocrinology and Diabetes, Fiona Stanley Hospital, Perth, WA 6150, Australia

**Keywords:** osteocalcin, undercarboxylated osteocalcin, GPRC6A, insulin resistance, diabetes, cardiovascular disease, bone, muscle

## Abstract

Recent advances have indicated that osteocalcin, and in particular its undercarboxylated form (ucOC), is not only a nutritional biomarker reflective of vitamin K status and an indicator of bone health but also an active hormone that mediates glucose metabolism in experimental studies. This work has been supported by the putative identification of G protein-coupled receptor, class C, group 6, member A (GPRC6A) as a cell surface receptor for ucOC. Of note, ucOC has been associated with diabetes and with cardiovascular risk in epidemiological studies, consistent with a pathophysiological role for ucOC in vivo. Limitations of existing knowledge include uncertainty regarding the underlying mechanisms by which ucOC interacts with GPRC6A to modulate metabolic and cardiovascular outcomes, technical issues with commonly used assays for ucOC in serum, and a paucity of clinical trials to prove causation and illuminate the scope for novel health interventions. A key emerging area of research is the role of ucOC in relation to expression of GPRC6A in muscle, and whether exercise interventions may modulate metabolic outcomes favorably in part via ucOC. Further research is warranted to clarify potential direct and indirect roles for ucOC in human health and cardiometabolic diseases.

## 1. Introduction—Osteocalcin and Undercarboxylated Osteocalcin 

The objectives of the present review are to highlight the role of osteocalcin, in particular its undercarboxylated form, as a hormone that regulates glucose homeostasis and cardiometabolic risk, by summarizing the available experimental and human evidence. Additionally, the emerging evidence implicating undercarboxylated osteocalcin in muscle function and glucose uptake, and its therapeutic potential for addressing cardiometabolic diseases are discussed.

Osteocalcin is a 49 amino acid polypeptide protein with a 5.7 KDa molecular weight. It is also known as gamma-carboxyglutamic acid-containing protein or bone gla protein [1]. Osteocalcin is the most abundant non-collagenous protein within the bone matrix, primarily produced by osteoblasts during the late stage of their differentiation [2]. However, the exact role of osteocalcin in the control of bone matrix formation, mineralization or maintenance is not fully understood [3]. In osteoblasts, after protein translation at the endoplasmic reticulum, osteocalcin undergoes carboxylation at the 17, 21 and 24 glutamic acid (Glu) residues by γ-glutamyl carboxylase. This process is facilitated by vitamin K. This post-translational modification changes the conformation of the osteocalcin protein, resulting in an increased affinity for the calcium ions exposed at the surface of the hydroxyapatite crystal in the bone matrix [2,4]. However, not all osteocalcin in bone is fully carboxylated, with a small amount of osteocalcin still containing one (mostly Glu17) or more empty Glu residue(s), collectively denoted as undercarboxylated osteocalcin (ucOC) [5].

Osteocalcin in the circulation is a marker of bone turnover [4]. Of the total amount of osteocalcin that is released into the circulation, a substantial proportion (40–60%) is ucOC, the amount of which is sensitive to vitamin K intake [3,5,6,7]. As such, a higher percentage of ucOC is a marker for vitamin K status, generally indicative of lower vitamin K availability [8,9,10]. Furthermore, a higher percentage of ucOC has been associated with increased risk of bone fracture in older adults, particularly women [11,12,13]. Therefore, the detection of circulating ucOC has long been recognized as having clinical predictive value as a nutritional biomarker and indicator of fracture risk. In addition, recent studies have illuminated a role for ucOC to regulate insulin secretion by beta cells and adiponectin by fat cells [14,15].

## 2. Osteocalcin, Glucose Homeostasis and GPRC6A—Experimental Studies 

### 2.1. Osteocalcin, Undercarboxylated Osteocalcin and Glucose Metabolism

It has been hypothesized that bone may be involved in energy regulation to ensure the energy supply needed during bone remodeling, which encompasses the cellular machinery responsible for maintaining the integrity of bone composition, structure and strength [14]. In 2007, the Karsenty group were the first to demonstrate that circulating ucOC regulates whole-body energy metabolism via mechanisms that involved increasing insulin secretion from pancreatic beta cells, as well as enhancing insulin sensitivity in peripheral tissues [15]. In this research, enteroccocal surface protein (*Esp*)-knockout mice (*Esp*^−/−^) lacking an osteoblast-expressed receptor-like protein tyrosine phosphatase (OST-PTP) exhibited increased β-cell proliferation, increased insulin and reduced glucose concentrations, and were protected from developing a diabetic phenotype [15]. Of note, this phenotype was completely reversed by the deletion of a single allele of the osteocalcin gene (*Ocn*), resulting in mice, which had a higher proportion of circulating osteocalcin in the undercarboxylated form and implicating osteocalcin as a regulator of glucose homeostasis. *Ocn*-knockout mice did not display a distinct bone phenotype, but later in life became glucose intolerant and obese with reduced pancreatic β-cell expression and insulin content [15]. Furthermore, administration of exogenous ucOC to wild-type mice improved insulin production and sensitivity supporting ucOC as the metabolically active form of osteocalcin [16,17]. Since then, independent research groups have confirmed that ucOC is involved in energy metabolism, however, the level of involvement is still not clear [18]. One group has reported that knockout of the osteocalcin gene in rats using the CRIPR/Cas9 method did not affect body weight or fasting glucose concentrations [19]. Recent experimental studies have implicated ucOC in muscle growth [20], male fertility [21], brain development and cognition [22], and anti-tumour immunity [23].

### 2.2. GPRC6A as a Putative Receptor for Undercarboxylated Osteocalcin

In keeping with an endocrine role for ucOC, G protein-coupled receptor, class C, group 6, member A (GPRC6A) has been identified by several groups as a putative receptor of ucOC [24,25,26]. GPRC6A belongs to the C family of G protein-coupled receptors and is widely expressed in cells of a number of human tissues such as skeletal muscle, brain, lung, liver, heart, kidney, pancreas, placenta, spleen, ovary, testis, prostate, leukocytes, and monocytes [25]. Expression of GPRC6A has also been documented in mouse muscle [20,27]. It has been shown that either global or tissue-specific knockout of GPRC6A leads to the loss of endocrine functions of ucOC in mice [21,24]. Furthermore, several studies have reported that the deficiency of GPRC6A in mouse tissues or cultured cells results in the absence of ucOC-induced activation of downstream signaling pathways [28,29,30,31]. By using computational docking models, Min Pi et al., [32] predicted that the *C*-terminal hexapeptide of ucOC docks to the extracellular side of the transmembrane domain of GPRC6A. Consistently, they also showed that the mutation of a computationally-identified binding site of GPRC6A decreased the activation of this receptor by ucOC *C*-terminal hexapeptide [32]. It is worth noting, however, that to date there is still no evidence of direct binding between ucOC and GPRC6A, thus it remains possible that GPRC6A only functions as a crucial upstream regulator, rather than the direct receptor, in the ucOC-triggered signaling cascade. Moreover, consensus on the role of GPRC6A in ucOC signaling is lacking, as some studies have shown that in vitro treatment of ucOC failed to activate GPRC6A that was exogenously expressed in CHO or HEK273 cells [33,34]. Therefore, whether GPRC6A is the receptor by which ucOC exerts endocrine functions in vivo requires further investigation.

### 2.3. Summary

- Undercarboxylated osteocalcin regulates insulin secretion and sensitivity in mice
- A G protein-coupled receptor GPRC6A is implicated in the actions of undercarboxylated osteocalcin in experimental studies

## 3. Osteocalcin, Diabetes and Cardiovascular Risk—Clinical and Epidemiological Studies

### 3.1. Osteocalcin, Metabolic Syndrome and Diabetes Risk

Clinical and epidemiological studies have examined associations of circulating osteocalcin with endpoints related to metabolic syndrome and diabetes (Table 1). Generally these were cross-sectional analyses assessing total circulating osteocalcin which have associated lower osteocalcin concentrations with higher risk of Type 2 diabetes [35,36,37], higher body mass index (BMI) and plasma glucose concentrations [37,38], lower insulin sensitivity [39,40]. In a large epidemiological study of 2493 men and women, lower osteocalcin concentrations were associated with higher BMI, fasting glucose and insulin resistance [41]. In another large epidemiological study of 2765 older men, lower osteocalcin concentrations were associated with higher waist circumference, glucose and triglyceride concentrations, and insulin resistance [42]. In both of those studies, adults with lower osteocalcin concentrations had a higher risk of having metabolic syndrome [41,42]. Subsequent studies have largely confirmed these findings, reinforcing the association of lower osteoalcin concentrations with insulin resistance and with metabolic syndrome in men and in postmenopausal women [43,44,45,46,47,48,49,50,51,52]. In keeping with these studies, a meta-analysis of observational studies confirmed that circulating total osteocalcin concentrations were lower in adults with metabolic syndrome or Type 2 diabetes [53]. Therefore there are ample observational studies in humans which support a pathophysiological role for osteocalcin in regulation of glucose metabolism and diabetes risk in vivo. However, there are two major limitations of these studies: firstly, causality cannot be inferred from cross-sectional analyses and one longitudinal analysis did not show an association of osteocalcin with incidence of Type 2 diabetes [46]. Secondly, data on circulating ucOC which is postulated to be the metabolically active form binding to GPRC6A were not reported in those studies (Table 1). 

### 3.2. Measuring Circulating Undercarboxylated Osteocalcin Concentrations

Standard immunoassays for osteocalcin (Table 1) recognise both carboxylated and undercarboxylated forms, giving a result for total circulating osteocalcin. Currently two methods are used to measure circulating ucOC concentrations. The hydroxyapatite (HAP) binding assay uses HAP to bind carboxylated osteocalcin which is then removed by centrifugation, after which ucOC is measured by immunoassay in the serum supernatant [54]. While this method should identify the metabolically active form of osteocalcin [15], it is technically more involved than a single-step immunoassay. Direct immunoassays use antibodies against ucOC; however, a commonly used commercially available ucOC antibody (Takara Shuzo Co., Kyoto, Japan) overestimates large ucOC fragments that can lead to inaccuracies in determination of ucOC or the ratio of ucOC to total osteocalcin [54]. Furthermore, this ucOC antibody might not reliably detect ucOC when uncarboxylated only at the Glu-17 position [55,56]. Therefore measurement of ucOC is not straightforward and frequently used methods do not distinguish the number and position of uncarboxylated Glu residues, and these limitations need to be considered in the interpretation of results. Compared to studies reporting total osteocalcin concentrations (Table 1), clinical and epidemiological studies, which have examined associations of ucOC with endpoints related to metabolic syndrome and diabetes tend to be smaller in size, at least in part due to the added requirement for assay of ucOC (Table 2).

### 3.3. Studies Associating Undercarboxylated Osteocalcin with Diabetes Risk in Men

A number of studies have reported ucOC results in relation to metabolic outcomes (Table 2). Several cross-sectional studies in men have documented associations of ucOC with glucose metabolism in vivo [57,63,70]. Two studies are of particular interest. A large epidemiological study of 1597 men aged ≥65 years found that ucOC was inversely associated with measures of glycemia and insulin resistance, remaining so after adjustment for total osteocalcin [63]. Another large epidemiological study, the Western Australian Health In Men Study (HIMS), analysed 2966 men aged ≥70 years in whom ucOC was assayed using a HAP-binding assay and also examined total osteocalcin and two non-osteocalcin markers of bone turnover, *N*-terminal propeptide of type I collagen (P1NP) and collagen type I *C*-terminal cross-linked telopeptide (CTX) [70]. That HIMS analysis found that all four bone turnover markers were inversely associated with diabetes risk; however, when all four were included in a multivariate model that included adjustment for conventional risk factors, ucOC remained robustly associated with diabetes risk while total osteocalcin and P1NP were no longer associated and the association of CTX was attenuated [70]. These epidemiological findings support the concept of ucOC being a predictor of diabetes risk in older men, distinct from total osteocalcin or other bone turnover markers. In a longitudinal study, an increase in ucOC was associated with a reduction in insulin resistance in men over an interval of two years [62]. 

### 3.4. Other Studies of Undercarboxyated Osteocalcin and Glucose Metabolism

In a study of 348 non-diabetic men and women, ucOC measured using a HAP-binding assay was not associated with insulin sensitivity at baseline; however, a lower % of ucOC predicted greater increase in insulin resistance at follow-up [58]. In a study of 129 overweight postmenopausal women without diabetes, a greater ratio of carboxylated to total osteocalcin correlated inversely with insulin sensitivity, supporting in an indirect manner a role for ucOC in regulation of glucose metabolism in women [56]. One study in 63 overweight or obese adults found an association of ucOC with β cell responses only in the subset of adults with impaired fasting glucose but not in those with normal glucose tolerance [67]. In studies of overweight men, ucOC was inversely correlated with fasting glucose and HbA1c, and both ucOC and the ratio of ucOC to total osteocalcin increased following exercise [61,68]. 

### 3.5. Studies of Undercarboxylated Osteocalcin in Adults with Diabetes

Several studies of ucOC have been reported in adults with Type 2 diabetes (Table 2). A case-control analysis of 153 adults with newly diagnosed Type 2 diabetes and 306 controls reported an association of both carboxylated osteocalcin and ucOC with risk of diabetes [66]. While some studies have not shown strong associations of ucOC with indices of glycemia [59,64] other studies have supported a role for ucOC as a marker for better indices of glycemia in adults with Type 2 diabetes [60,71]. Of note, while these studies relate ucOC to insulin sensitivity and Type 2 diabetes, ucOC does not appear to be associated with indices of glycemia in the setting of gestational diabetes [69] and the role of ucOC in Type 1 diabetes remains uncertain [65,72]. Therefore, there is observational data supporting a role for ucOC to modulate risk of Type 2 diabetes, particularly in older men. The role of ucOC to modulate glucose metabolism once Type 2 diabetes is already present is less clear. 

### 3.6. Osteocalcin and Cardiovascular Risk

Diabetes has long been recognised as a cardiovascular risk factor, whose presence is predictive of cardiovascular events and mortality [73,74,75]. Therefore, given the experimental and epidemiological evidence linking osteocalcin and ucOC with diabetes risk, the next question is whether or not osteocalcin and in particular ucOC by modulating cardiometabolic health influence the incidence of cardiovascular events and mortality. Several epidemiological studies have examined associations of osteocalcin with outcomes related to cardiovascular events and mortality (Table 3). In a cross-sectional study of 461 adults undergoing coronary angiography, those with proven coronary artery disease had lower total circulating osteocalcin as a group [76]. A longitudinal study of 781 men found no association of total osteocalcin with mortality [77]. In a longitudinal analysis from HIMS of 3542 men aged ≥70 years, there was a U-shaped association of total osteocalcin with all cause and cardiovascular mortality [78]. Subsequent studies have reported associations of higher baseline total osteocalcin with lower all-cause mortality, and lower baseline total osteocalcin with all-cause and cardiovascular mortality in men [79,80], and a U-shaped association of total osteocalcin with non-cardiovascular but not all-cause or cardiovascular mortality in women [81]. Therefore, on balance, lower total osteocalcin appears to be a predictor of all-cause and cardiovascular mortality in men, while additional research is needed to clarify the prognostic implications of high total osteocalcin concentrations in men. The association of total osteocalcin with mortality in women is less clear. However, these studies are limited by the lack of ucOC data.

### 3.7. Circulating Undercarboxylated Osteocalcin and Cardiovascular Risk

A cross-sectional analysis involving 114 men reported an association of higher ucOC and an increased ucOC/total osteocalcin ratio with coronary artery calcification [82]. The significance of this finding, however, is uncertain as the sample size was relatively small, the cross-sectional nature of the analysis precludes attribution of causation and no association was seen in women. Of note, a more recent longitudinal analysis from HIMS of 3384 men aged 70–89 years followed for seven years demonstrated an association of higher ucOC/total osteocalcin ratio with lower incidence of myocardial infarction after adjusting for conventional cardiovascular risk factors [83]. In HIMS, the ratio of ucOC to total osteocalcin was not predictive of stroke, suggesting a differential influence on distinct vascular territories. Those findings support the concept that the proportion of circulating ucOC functions as a biomarker for incidence of myocardial infarction. The possible underlying mechanisms and whether interventions that result in a greater proportion of circulating ucOC would reduce cardiovascular risk remain to be elucidated.

### 3.8. Matrix Gla Protein and Cardiovascular Risk

Of note, matrix γ-carboxyglutamic acid (Gla) protein (MGP) is a peptide associated with the organic phase of bone and cartilage, which shares substantial homology with osteocalcin [4]. MGP is expressed in chondrocytes and vascular smooth muscle, and acts as an inhibitor of vascular calcification [84,85]. MGP undergoes γ-carboxylated at Glu residues under the influence of vitamin K and uncarboxylated forms of MGP in the circulation have been associated with arterial stiffness in adults, and with peripheral arterial calcification and incidence of cardiovascular events in patients with Type 2 diabetes [86,87,88]. However, it is unclear whether or not there is an analogous role for ucOC to modulate vascular calcification and thereby risk of cardiovascular events.

### 3.9. Summary

- Epidemiological studies associate lower circulating total osteocalcin concentrations with insulin resistance and risk of Type 2 diabetes
- Methods for assay of circulating undercarboxylated osteocalcin need to be optimised
- Epidemiological studies associate lower circulating concentrations of undercarboxylated osteocalcin, or a lower ratio of undercarboxylated to total osteocalcin, with risk of Type 2 diabetes and incidence of myocardial infarction

## 4. Osteocalcin, Exercise and Muscle Function—Experimental Studies

### 4.1. Putative Interaction of Bone, Muscle and Metabolism

The initial discovery of a metabolic role for ucOC focused attention on insulin sensitivity and diabetes risk [15,16,17]. Whilst these initial studies did not focus primarily on muscle, it should be noted that skeletal muscle plays a major role in glucose uptake and utilization [89,90]. It is a major site for nutrient storage and energy expenditure [91,92]. In keeping with the importance of skeletal muscle in glucose metabolism, increasing interest has focused on the link between bone, muscle and metabolism, via a potential role of ucOC acting in muscle [93]. 

### 4.2. The Role of Undercarbxoylated Osteocalcin in Skeletal Muscle: Evidence from Mouse Models

Evidence thus far has indicated that ucOC may participate in the endocrine modulation of skeletal muscle functions, via the GPRC6A receptor and its downstream signaling pathways in muscle cells [28]. In vivo studies in mice first pointed to ucOC effects in skeletal muscle insulin sensitivity and signalling. These studies reported an association between higher serum ucOC levels due to loss of *Esp*, *activating transcription factor 4 (Atf4)* or *the class O of forkhead box transcription factor 1 (Foxo1)* expression in osteoblasts with enhanced expression of insulin target genes in skeletal muscle [94,95]. A further study showed higher insulin-stimulated muscle glucose uptake in *Esp*^−/−^ mice compared to wide type (WT) littermates. In muscle from mice subject to a high fat diet (HFD), administration of ucOC reduced the deleterious effect of the HFD diet on gene expression and endoplasmic reticulum stress, fat accumulation, and autophagy [96,97]. It should be noted, however, that ucOC was also able to enhance the secretion of insulin from the pancreas [15,16], adiponectin from adipose tissue [16], and glucagon-like peptide-1 (GLP-1) from the intestines [98,99]. Thus, the insulin-sensitising effects of ucOC on muscle indicated by in vivo studies may be affected by indirect factors rather than representing a purely direct.

### 4.3. The Role of Undercarboxylated Osteocalcin in Skeletal Muscle: Cell and Tissue-Based Studies

Other studies have been able to elucidate some of the direct effects of ucOC on muscle cells by using in vitro and isolated (exercised) mouse muscle models. In C2C12 myotubes, a subclone model or rapidly differentiating mouse myotubes, ucOC exposure at physiological concentrations dose-dependently augmented insulin-stimulated glucose uptake [27,100]. Ex vivo, a 2.5-h ucOC treatment (30 ng/mL) enhanced muscle glucose uptake in insulin-stimulated mouse soleus muscles, but not in insulin-stimulated extensor digitorum longus (EDL) muscles [101]. It should be noted that soleus and EDL muscles are made of distinct fiber-type compositions. While soleus primarily consists of slow twitch type I and EDL of fast twitch type II fibers, respectively, this result indicates that ucOC possibly favors muscle insulin sensitivity in a fiber type-specific manner [101]. Importantly, ucOC also triggers non-insulin-stimulated glucose uptake [102]. Therefore, further investigations into the mechanisms by which ucOC exert direct effects on muscle insulin sensitivity are warranted.

### 4.4. Exercise and Insulin Sensitivity: A Role for Undercarboxylated Osteocalcin?

The benefits of exercise on muscle energy metabolism and insulin sensitivity have been well documented [103,104]. Even a single session of exercise improves insulin sensitivity for up to 48 h following exercise [105,106]. An important and often overlooked benefit of exercise is that acute exercise enhances glucose uptake in skeletal muscle in a pathway independent of insulin, meaning people with insulin resistance and Type 2 diabetes may have normal glucose uptake in skeletal muscle during and for a period following exercise [106,107,108]. The exact mechanism by which this interesting phenomenon occurs remains unclear. Some reports have suggested that this post-exercise effect on glucose uptake may be attributed to the alteration of humoral factors, one of which may indeed be ucOC [61,68,106,109].

### 4.5. Exercise as a Stimulus for Increased Circulating Undercarboxylated Osteocalcin

Due to the specialized functions of bone cells, namely osteoblasts, osteoclasts and osteocytes, bone is a dynamic tissue that responds to mechanical loading and unloading by modifying its mass and strength via cellular driven remodeling [110]. It is thus possible that exercise, which stresses the bone via increased mechanical loading, would have an effect on markers of bone remodeling, including osteocalcin and ucOC [111,112]. Reports of the effect of exercise on total osteocalcin are contentious, with some researchers describing increases in total osteocalcin concentrations with acute exercise [113,114], while others reported lower [115], or unchanged [116,117] total osteocalcin concentrations shortly after exercise. The existence of these contradictory data may be due to different exercise modes or intensities, or different time points of blood sampling during/following exercise [111,118]. However, total osteocalcin is not the same as uOC and overall, studies seem to agree that acute exercise associates with higher circulating ucOC concentrations. In humans, ucOC concentrations were reported to be increased by 6–14% immediately after a single session of high intensity aerobic exercise [61,68]. In mice, a much higher increase, up to 2.5-fold, was observed during and shortly after a single bout of aerobic treadmill running [28]. 

### 4.6. Exercise, Undercarboxylated Osteocalcin and Insulin Sensitivity

Increases in serum ucOC concentrations following exercise may be clinically important. As exercise improves insulin sensitivity and glycaemic control, it is likely that the increase in ucOC may contribute to the insulin-sensitizing effect of exercise. Indeed, it was reported that a single session of high-intensity exercise (95.1% ± 1.9% of HRpeak) by obese men increased circulating ucOC, which correlated with enhanced insulin sensitivity following exercise [61,68]. Furthermore, analyses from muscle biopsies revealed ucOC levels were also associated with higher insulin signaling activity in the vastus lateralis muscle following exercise [68]. In an ex vivo mouse study where isolated muscles were loaded into specialized contraction baths, ucOC treatment was reported to enhance insulin-stimulated glucose uptake in EDL muscles post-ex vivo contraction, indicating a direct role for ucOC in muscle insulin-sensitizing effect following contraction-stimulated exercise [27]. These studies suggest a potential role for exercise as a stimulus to increase circulating ucOC, which may then, in turn, modulate some of the beneficial effects of exercise. It should be noted that the exercise-induced ucOC increase was usually transient [61,68], thus whether and how this short-term ucOC enhancement contributes to muscle insulin sensitivity hours after exercise requires further exploration.

### 4.7. Undercarboxylated Osteocalcin and Muscle Strength

Mice lacking osteocalcin were reported to have lower muscle mass and volume compared to their wild-type counterparts of a similar age [28]. Moreover, administration of ucOC increased muscle mass in older mice [20]. Decreased ucOC levels were associated with reduced muscle mass and strength, in both EDL and soleus muscles, in rats with disuse atrophy induced by hind limb immobilization [119]. In cultured C2C12 myoblasts treatment with ucOC increased proliferation and differentiation, effects that were mediated, at least in part, via GPRC6A [29]. Despite these animal and cellular data suggesting an intrinsic role for ucOC in muscle function, human data are limited. In a cross-sectional study of women aged ≥70 years in which ucOC was assayed using a HAP-binding assay, a positive correlation was found between % ucOC and quadriceps muscle strength [120]. In an interventional study of 62 patients with hypoparathyroidism, treatment with recombinant parathyroid hormone (rPTH) resulted in an increase in % ucOC which was not associated with energy metabolism [121], but was positively associated with the change of maximum force generated in elbow extension [122]. However, in that study, the increase in ucOC was not associated with other tests of muscle and physical performance [122]. Therefore, while there is some data to support a role for ucOC in the regulation of muscle strength and function, in addition to its role in muscle glucose uptake and metabolism, interventional studies are needed to determine if there is a causative effect and whether there is scope for novel therapeutic approaches to enhance both glucose metabolism and muscle function.

### 4.8. Molecular Mechanisms Underlying Undercarboxylated Osteocalcin’s Role in Insulin Resistance

The mechanisms underlying the action of ucOC in target tissues to improve insulin secretion and sensitivity are not the major focus of the current review and as such are not described in detail. However, substantial emerging evidence suggests that relevant molecular mechanisms include activation of the ERK MAPK pathway in β cells [31,32] resulting in increased cell proliferation and insulin secretion, as well as increased insulin sensitivity in peripheral tissues via the activation of the PI3K/Akt pathway [20,96], the cAMP/CREB pathway [20], the AS160 protein [27,101], and the downregulation of the ER stress pathway [96,97].

### 4.9. Osteocalcin and Ageing: Relevance to Muscle Mass and Glucose Homeostasis

In cross-sectional studies of men and women, circulating osteocalcin is highest in early adulthood, lower in mid-life, and higher again in older age [123,124,125]. How the proportion of ucOC compared with total osteocalcin changes during ageing is less clear. In mice, there is a substantive decrease in total circulating osteocalcin and ucOC concentrations between the ages of 2 and 9 months, and muscle-specific deletion of *Gprc6a* reduced exercise capacity in young mice [28]. Interestingly, administration of exogenous osteocalcin restored the exercise capacity of 12 and 15-month old mice to levels comparable with 3-month old mice [28]. The human correlate is the emergence of sarcopenia and frailty in older age, linked with an increasing incidence and prevalence of diabetes [126]. In a study of men aged 79–97 years, those with diabetes had poorer physical performance demonstrating the inter-relationship between ageing, deterioration of physical function and diabetes [127]. Whether ucOC might ameliorate age-related changes in muscle and other tissues to restore physical function and improve glucose homeostasis in humans remains to be seen.

### 4.10. Summary

- Undercarboxylated osteocalcin enhances muscle glucose uptake in mice and cells
- Undercarboxylated osteocalcin increases muscle mass in older mice and increases proliferation of muscle cells
- Exercise increases circulating undercarboxylated osteocalcin and insulin sensitivity in vivo

## 5. Discussion and Conclusions

Our understanding of the interaction between bone and energy metabolism has been improved by the identification of an endocrine role for ucOC in the modulation of insulin secretion and sensitivity, and therefore risk of Type 2 diabetes. While the initial discoveries were made in experimental mouse and cellular (in vitro) models, these were rapidly translated via observational clinical and epidemiological studies that supported the relevance of osteocalcin, and particularly ucOC, to glucose metabolism and diabetes risk in vivo. These studies have extended our understanding of ucOC, or the ratio of ucOC to total osteocalcin, as predictors not only of indices of insulin resistance and risk of Type 2 diabetes, but also of cardiovascular risk and incidence of myocardial infarction. Furthermore, the role of ucOC in muscle function and the effects of exercise to increase circulating ucOC in vivo illuminate new mechanisms by which exercise, bone and muscle are able to interact to regulate energy metabolism. While GPRC6A has been postulated as the putative receptor for ucOC, including in muscle, much more work is needed to clarify whether this is the primary route by which ucOC exerts its actions in different tissues. 

At this stage, interventional studies that prove a causal role for ucOC in the reduction of diabetes and cardiovascular risk in humans are lacking. Anti-resorptive therapies for osteoporosis generally reduce bone turnover markers and randomised controlled trials of these agents have not reported differences in the incidence of diabetes in treatment vs control arms [128]. However, those trials were never designed with incidence of diabetes as an outcome. By contrast, a retrospective analysis of a large cohort of primary care patients prescribed anti-resorptive therapy with bisphosphonates and practice-matched unexposed persons associated exposure to bisphosphonates with reduced risk of Type 2 diabetes [129]. Neither of these studies reported ucOC results [128,129]. 

In studies involving men and women, dietary intake of vitamin K1 (phylloquinone) was associated with insulin sensitivity [130], while dietary intakes of either vitamin K1 or K2 (menaqionones) were associated with lower risk of Type 2 diabetes [131,132]. In another study, vitamin K2 intake was associated with lower occurrence of metabolic syndrome [133]. However, a higher intake of vitamin K might reflect better nutritional quality of the diet and greater engagement in healthy lifestyle behaviors overall, for example, consumption of leafy green vegetables which is the main source of vitamin K1 [134]. Clinical trials of vitamin K supplementation have yielded mixed results. In a four-week trial in premenopausal women at risk of diabetes, vitamin K1 supplementation resulted in lower 2-h glucose concentrations and improved insulin sensitivity [135]. By contrast, a 12-month study of vitamin K1 supplementation in postmenopausal women showed no change in indices of glucose homeostasis [136]. A large randomized placebo-controlled trial in 355 older men and women of 36 months supplementation with vitamin K1 found less progression of insulin resistance in men, but not in women [137]. A four-week study of vitamin K2 supplementation in healthy young men reported increased insulin sensitivity [138]. However, studies of vitamin K2 supplementation in young men and women, and in postmenopausal women, have found no effect on adiponectin [139,140]. A systematic review of eight trials involving 1077 participants reported no effect of vitamin K supplementation on insulin sensitivity, concluding that further well-designed randomized controlled trials with large sample sizes are needed [141]. Vitamin K supplementation consistently reduced ucOC, increasing the proportion of carboxylated osteocalcin [135,136,138,139,140]. Thus, the overall effect of vitamin K supplementation on indices of glucose metabolism remains to be fully clarified, reflecting the broader actions of vitamin K1 and K2 on pathways distinct from osteocalcin [141,142,143,144]. 

Dedicated interventional studies are essential to determine whether increasing circulating ucOC or the ratio of ucOC relative to total osteocalcin would improve insulin sensitivity and thus reduce the risk of Type 2 diabetes in humans. Administering recombinant ucOC has been informative in mice [16,17], but this approach is logistically more challenging in humans and as yet there have been no clinical trials of ucOC to determine its effects on diabetes and cardiovascular risk. Increasing circulating ucOC by exercise offers an alternative interventional approach, although care would be required in the interpretation of such studies when distinguishing the effects of exercise versus the effects of ucOC in the pancreas, fat and muscle tissues. Thus, the endocrine role of ucOC, linking as it may muscle, bone and metabolism to influence diabetes and cardiovascular risk, remains a key arena for future research to improve human health. 

In terms of future perspectives and research directions, additional mechanistic studies to clarify the mechanisms by which ucOC exerts its actions in various tissues, including β-cells and muscle would be highly valuable. Further work is needed to ascertain whether GPRC6A is a major receptor for ucOC in these and other tissues, bearing in mind that another G-protein coupled receptor Gpr158 has been found to mediate the effect of osteocalcin on cognition in mice [145]. Epidemiological studies utilizing robust assays for circulating ucOC will continue to provide important information regarding the associations of ucOC in humans. The interaction of ucOC, muscle function and glucose homeostasis is a key area further research, with exercise interventions representing a possible pathway to manipulate ucOC with the ultimate goal of improving cardiometabolic health.

## Figures and Tables

**Table 1:** Selected studies examining associations of total circulating osteocalcin (TOC) with insulin resistance, metabolic syndrome and diabetes in men and women are summarised. X = cross-sectional analysis, L = longitudinal analysis, I = interventional component, HOMA = homeostasis model assessment, HOMA-B = β-cell activity, HOMA-IR = insulin resistance.

First Author, Year [ref no.]	Study (Type)	Results
Im J-A, 2008 [35]	339 post-menopausal women, 31 with Type 2 diabetes (X)	Serum TOC was lower in women with Type 2 diabetes vs. controls (17.5 vs. 22.2 μg/L), and correlated inversely with HbA1c (*r* = −0.22) and IR (*r* = −0.16)
Zhou M, 2009 [36]	254 men (128 newly diagnosed Type 2 diabetes) and 180 postmenopausal women (92 with diabetes) (X)	Serum TOC was lower in adults with Type 2 diabetes vs. controls (15.1 vs. 16.8 μg/L).
Kindblom JM, 2009 [37]	857 non-diabetic and 153 diabetic men (X)	Diabetic men had lower TOC (21.7 vs. 27.8 μg/L), TOC was inversely related to body mass index (BMI), fat mass and fasting glucose.
Kanazawa I, 2009 [38]	179 men and 149 post-menopausal women with Type 2 diabetes (X)	TOC correlated negatively with fasting plasma glucose (*r* = −0.24 for men, −0.19 for women) and HbA1c (*r* = −0.16, −0.27). TOC correlated with total adiponectin in women (*r* = 0.30).
Fernandez-Real JM, 2009 [39]	149 non-diabetic men (X), and 46 non-diabetic men and women (I)	Serum TOC correlated with insulin sensitivity (*r* = 0.23), and total adiponectin (*r* = 0.19). TOC was increased by dietary weight loss (16.8% of body weight) or weight loss (8.7%) + exercise.
Pittas AG, 2009 [40]	380 men and women (X), 198 (L), 5% with diabetes	Serum TOC inversely correlated with fasting glucose, insulin and IR. Higher TOC associated with lower rise in fasting glucose over 3 years.
Saleem U, 2010 [41]	2493 men and women (X)	Serum TOC inversely correlated with BMI, fasting glucose, IR and leptin, positively correlated with adiponectin. TOC in highest quartile associated with reduced odds of metabolic syndrome.
Yeap BB, 2010 [42]	2765 older men with metabolic syndrome present in 797 (28.8%) (X)	TOC level was inversely associated with waist circumference, glucose, triglyceride levels and IR, and was lower in men with metabolic syndrome (20.1 vs. 21.4 μg/L). Men with TOC of 13.3–16.6 and <13.3 μg/L had 1.5 to 2-fold increased risk of metabolic syndrome compared to men with TOC ≥ 30 μg/L.
Tan A, 2011 [43]	2344 men aged 20–69 years (X)	TOC correlated with HDL and was inversely associated with BP, glucose, triglycerides, waist circumference and BMI. Men with TOC in the lowest quartile had a higher odds ratio for having metabolic syndrome.
Bao Y, 2011 [44]	181 men who underwent coronary angiography (X)	TOC was lower in men with metabolic syndrome. In a subgroup of 60 men with normal glucose tolerance men with multi-vessel coronary artery disease had lower TOC compare to men without coronary artery disease.
Bae SJ, 2011 [45]	567 men and postmenopausal women (X)	TOC was lower in postmenopausal women with metabolic syndrome (18.9 vs. 22.5 ug/L) and in men with metabolic syndrome (14.6 vs. 16.1 ug/L) compared to those without metabolic syndrome.
Lee SW, 2012 [46]	214 postmenopausal women (X)	TOC was not associated with fasting glucose, but was inversely associated with HOMA-IR
Movahed A, 2012 [47]	382 postmenopausal women (X)	Lower TOC was associated with higher odds ratio of having Type 2 diabetes
Hwang Y-C, 2012 [48]	1229 men aged 25–60 years without diabetes at baseline, of which 90 developed Type 2 diabetes during mean follow-up of 8.4 years (L)	Baseline TOC in tertiles was inversely associated with HOMA-IR in cross-sectional analysis, but was not associated with incident Type 2 diabetes in longitudinal analysis.
Oosterwerff MM, 2013 [49]	1284 persons (629 men and 655 women) aged 65–88 years (X)	TOC was inversely associated with metabolic syndrome with odds ratio 3.7 for those with TOC in the lowest compared to the highest quartile of values.
Yang R, 2013 [50]	1789 postmenopausal women aged 41–78 years (X)	TOC was lower in women with metabolic syndrome (18.5 vs. 21.1 ug/L) compared to those without. Women with higher TOC had lower odds ratio for metabolic syndrome.
Confavreux CB, 2014 [51]	798 men aged 51–85 years (X)	Higher TOC was associated with lower odds ratio for metabolic syndrome.
Kang J-H, 2016 [52]	98 persons (24 men and 74 women) mean age 53.5 years (X)	TOC was inversely associated with fasting glucose and HOMA-IR, but not with atherosclerotic plaque in the subset of 31 persons who had coronary CT angiography.

**Table 2:** Selected studies examining associations of undercarboxylated osteocalcin (ucOC) with insulin resistance, metabolic syndrome and diabetes in men and women are summarised. X = cross-sectional analysis, L = longitudinal analysis, I = interventional component, HOMA = homeostasis model assessment, HOMA-B = β-cell activity, HOMA-IR = insulin resistance, TOC = total osteocalcin, P1NP = *N*-terminal propeptide of type I collagen, CTX = collagen type I *C*-terminal cross-linked telopeptide. * ucOC assayed using hydroxyapatite binding, # ucOC assayed using ucOC antibody.

First Author, Year [ref no.]	Study (Type)	ucOC Assay	Results
Hwang Y-C, 2009 [57]	199 men (X)	#	Higher ucOC associated with greater insulin sensitivity (HOMA-B).
Shea MK, et al., 2009 [58]	348 non-diabetic men and women (M = 142, F = 206) (X, L)	*	Higher total and carboxylated OC levels (not ucOC) associated with insulin sensitivity, association attenuated by adjustment for adiponectin. Higher carboxylated OC level at baseline predicted less change in IR at 3 years, lower % ucOC predicted greater increase in IR.
Kanazawa I, 2009 [59]	50 men and women with poorly controlled Type 2 diabetes (L)	#	After one month of improved glycemic control, TOC level increased, ucOC was unchanged but the ratio of ucOC/TOC decreased.
Kanazawa I, 2011 [60]	180 men and 109 postmenopausal women with Type 2 diabetes (X)	#	ucOC was inversely correlated with fasting glucose and HbA1c in men, but not in postmenopausal women.
Levinger I, 2011 [61]	28 men aged 52.4 years with BMI 32.1 kg/m^2^ (X, I)	#	ucOC inversely correlated with fasting glucose and HbA1c, ucOC increased following aerobic and strength exercise.
Bullo M, 2012 [62]	79 men aged 55–80 years with cardiovascular risk factors (X, L)	#	Baseline ucOC was not associated with HOMA-IR at 2 years, change in ucOC was inversely associated with change in HOMA-IR over 2 years.
Iki M, 2012 [63]	1597 men aged ≥65 years (X)	#	TOC and ucOC were correlated (correlation coefficient 0.66). TOC and ucOC in quintiles were inversely associated with fasting glucose, HbA1c and HOMA-IR. Inverse association of ucOC with these outcomes remains significant after adjusting for TOC (but not vice versa). Higher quintiles of ucOC were associated with lower odds ratios for prevalent Type 2 diabetes (but not TOC).
Mori K, 2012 [64]	129 adults with Type 2 diabetes mean age 54.9 years (X)	#	ucOC was not associated with insulin resistance using euglycemic hyperinsulinemic clamp in adults with Type 2 diabetes
Thrailkill KM, 2012 [65]	115 adults with Type 1 diabetes and 55 controls mean age 18.8 years	#	No difference in ucOC between adults with Type 1 diabetes and controls. ucOC was inversely associated with HbA1c.
Diaz-Lopez A, 2013 [66]	153 adults with newly diagnosed Type 2 diabetes and 306 matched controls, mean age 66.3 years	#	Carboxylated osteocalcin (not ucOC) was inversely associated with HOMA-IR in cases, and fasting glucose in controls. Lower carboxylated osteocalcin or ucOC in tertiles were associated with higher odds ratio for incident diabetes.
Gower BA, 2013 [67]	63 overweight/obese adults with normal (*N* = 39) or impaired fasting glucose (*N* = 24) (X)	*	TOC was associated with insulin sensitivity in the whole cohort. ucOC was associated with indices of β cell response in the subset with impaired fasting glucose.
Levinger I, 2014 [68]	11 men aged 58.1 years with BMI 33.1 kg/m^2^ (I)	*	Exercise increased ucOC and ucOC/TOC ratio, reduced glucose concentrations and improved insulin sensitivity.
Saucedo R, 2015 [69]	60 women with gestational diabetes and 60 with normal glucose tolerance (X)	#	No difference in TOC or ucOC in women with gestational diabetes compared to women with normal glucose tolerance.
Yeap BB, 2015 [70]	2966 men aged ≥70 years (X)	*	Higher ucOC was associated with reduced diabetes risk (odds ratio per 1 SD increase after adjusting for conventional risk factors = 0.55). Similar results were seen for TOC, P1NP and CTX. When all 4 markers were included in the fully adjusted model, higher ucOC remained associated with reduced diabetes risk (odds ratio 0.56) while TOC was no longer associated.
Bonneau J, 2017 [56]	129 overweight/obese postmenopausal women without diabetes mean age 57.7 years (X)	#	Ratio of carboxylated to total osteocalcin correlated inversely with insulin sensitivity assessed using euglycemic hyperinsulinemic clamp and positively with HOMA-IR in postmenopausal women.
Takashi Y, 2017 [71]	50 adults with Type 2 diabetes mean age 59.2 years (X)	#	ucOC correlated with change in *C*-peptide following glucagon, and *C*-peptide response to eating a meal.
Yeap BB, 2017 [72]	108 adults with Type 1 diabetes mean age 39.1 years (X)	*	ucOC was not associated with fasting glucose, HbA1c or daily insulin dose in adults with Type 1 diabetes.

**Table 3:** Selected studies examining associations of total osteocalcin (TOC) and undercarboxylated osteocalcin (ucOC) with outcomes related to cardiovascular disease and mortality. X = cross-sectional study, L = longitudinal study, CHD = coronary heart disease. * ucOC assayed using hydroxyapatite binding, # ucOC assayed using ucOC antibody, N/A = no ucOC results reported.

First Author, Year [ref no.]	Study (Type)	ucOC Assay	Results
Szulc P, 2009 [77]	781 men aged ≥50 years (L)	N/A	TOC was not associated with mortality, while higher bone resorption markers were associated.
Yeap BB, 2010 [78]	3542 men aged 70–89 years followed for 5.2 years (L)	N/A	U-shaped association of TOC with all-cause and cardiovascular mortality.
Zhang Y, 2010 [76]	461 adults (243 with CHD and 218 without) undergoing coronary angiography (X)	N/A	TOC was lower in group with CHD.
Confavreux CB, 2013 [79]	774 men aged 51–85 years followed for 10 years (L)	N/A	Higher baseline TOC was associated with less progression of abdominal aortic calcification and lower all-cause mortality.
Lerchbaum E, 2013 [80]	2271 men referred for coronary angiography (L)	N/A	Association of TOC in lowest quintile with all-cause and cardiovascular mortality.
Lerchbaum E, 2014 [81]	986 women aged 58–72 years (L)	N/A	U-shaped association of TOC with non-cardiovascular mortality, TOC was not associated with all-cause or cardiovascular mortality.
Choi S-H, 2015 [82]	162 adults (114 men and 48 women) (X)	#	Higher ucOC and ratio of ucOC/TOC found in men with coronary artery calcification (no differences found in women).
Yeap BB, 2015 [83]	3384 men aged 70–89 years followed for 7 years (L)	*	Higher ratio of ucOC/TOC was associated with lower incidence of myocardial infarction, but was not associated with stroke.
